# Dielectric and Mechanical Properties of CTAB-Modified Natural Rubber Latex–Cement Composites

**DOI:** 10.3390/polym14020320

**Published:** 2022-01-13

**Authors:** Nutthakritta Phromviyo, Jakkree Boonlakhorn, Patcharapol Posi, Prasit Thongbai, Prinya Chindaprasirt

**Affiliations:** 1 Sustainable Infrastructure Research and Development Center, Department of Civil Engineering, Faculty of Engineering, Khon Kaen University, Khon Kaen 40002, Thailand; nutthaphrom@gmail.com; 2 Giant Dielectric and Computational Design Research Group (GD-CDR), Department of Physics, Faculty of Science, Khon Kaen University, Khon Kaen 40002, Thailand; jakkree_boonlakhorn@hotmail.com (J.B.); pthongbai@kku.ac.th (P.T.); 3 Department of Civil Engineering, Faculty of Engineering, Rajamangala University of Technology Isan Khon Kaen Campus, Khon Kaen 40000, Thailand; mister_wing@hotmail.com

**Keywords:** cement paste, natural rubber latex, cetyl trimethyl ammonium bromide, surfactant, mechanical property, dielectric properties

## Abstract

Cetyl trimethyl ammonium bromide (CTAB)-modified natural rubber latex/Portland cement paste (CTAB + NL/PC) composites were fabricated by varying the NL/cement and CTAB/cement ratios to improve the elastic property of PC. The stability and workability of the CTAB-modified NL particles in the PC matrix were significantly improved. The microstructure and dielectric property analyses of PC, CTAB/PC, NL/PC, and (CTAB + NL)/PC composites were performed to describe the interaction mechanism between the CTAB-modified NL and PC. The portlandite phase in PC was reduced by incorporating CTAB + NL. Although the tensile strength of NL/PC was significantly increased, its compressive strength also greatly decreased by ~40.3%. The tensile and compressive strengths of CTAB/PC were not significantly improved. Notably, the tensile strength of (CTAB + NL)/PC was significantly increased compared to those of PC, CTAB/PC, and NL/PC, while the depreciated compressive strength was only 18.7%. The optimized compressive–tensile performance of (CTAB + NL)/PC was equal to that of PC. The dielectric constants of NL/PC, CTAB/PC, and (CTAB + NL)/PC were reduced due to the low dielectric constant of NL and the ability of CTAB to capture negative charges in the PC matrix, leading to a reduction in the negative surface charges and hence the interfacial polarization. This result was confirmed by the decreased loss tangent in a low-frequency range, which is usually reduced by decreasing the free charges. This work provides a comprehensive guideline for significantly improving the elastic property of PC while retaining a high compressive strength.

## 1. Introduction

Cement materials, which have been used in the construction of buildings, are well known for their high compressive strength. However, they have a low tensile strength, toughness, flexural strength, and durability, which may be barriers to their use in unique modern structures such as those that can withstand high vibrations. To improve these disadvantages, several studies have focused on the modification of cemented materials with polymeric materials and fiber reinforcement [1,2,3,4,5]. Previous studies reported that fiber reinforcement would improve the tensile strength and flexural strength of cement materials [6,7,8,9,10].

In recent years, the incorporation of polymeric materials has been an important topic in the context of conventional fiber reinforcement. Styrene-butadiene rubber (SBR) and natural rubber latex (NL) have been widely used to improve the physical, mechanical, and durability properties of cement materials [11,12,13,14,15,16]. The usage of NL for cement paste, mortar, and concrete has significantly increased [17,18,19,20,21]. NL is classified as an environmentally friendly polymer elastomer latex material, which can be widely used to enhance the tensile properties of cement materials owing to its excellent flexibility, ductility, and toughness properties [18,22,23,24,25,26,27,28,29]. Unfortunately, when the cement and NL are mixed together, the workability is decreased due to their incompatibility. Therefore, the compressive strength and workability of NL/cement composites would decrease mainly as the amount of NL increases. To solve this problem, the addition of suitable surfactants into NL/cement-based composite materials can improve the compatibility between the matrix and filler [24,25,26,30]. The surfactant can modify the surface charge of NL particles, which can cause compatibility in the composites. Generally, after adding a surfactant, many air bubbles occur in the cement composite matrix, which requires adding an anti-foaming agent. Nevertheless, some anti-foaming agents are expensive and not environmentally friendly, which is unsuitable for use at a large scale for the construction of buildings. Therefore, the selection of the surfactants for NL/cement composites is very important.

To overcome the incompatibility between NL and cement, cetyl trimethyl ammonium bromide (CTAB) has been used as a surfactant admixture without an anti-foaming agent and a plasticizer admixture. Generally, CTAB is a cationic surfactant that is an effective antiseptic agent against bacteria and fungi [31,32,33]. Using CTAB as a surfactant not only improves the mixing compatibility, but also inhibits the bacteria and fungi in cement. Moreover, CTAB’s crosslink structure has been suggested to improve the stability of NL, which is modified and mixed with other materials [34,35]. The aim of this research work was to improve the compatibility between the cement and NL using a suitable surfactant and to describe the interaction mechanism between the cement and NL, which were combined with a surfactant, via dielectric spectroscopy.

Generally, the dielectric response in composites is primarily contributed by the accumulation of free changes at the internal interface, producing interfacial polarization [36,37,38]. Thus, variations in the dielectric constant (ε′) of heterogeneous dielectric materials may result from the changes in the free charges in the composites [36,39]. In this case, the interaction between the surfactant and free changes in the composites would result in decreased free charges, hence the variations in the dielectric constant. The reduced free changes can be reflected by the decrease in the loss tangent (tanδ) in a low-frequency range [40,41]. Furthermore, the physical and chemical characteristics of any parts in the composites can also affect the dielectric response in the composites.

In this work, low-cost materials that are environmentally friendly were selected as the starting raw materials to fabricate the NL/cement composites using a simple method without a foaming agent and plasticizer. The mechanical and dielectric properties of the CTAB-modified NL/cement composites were investigated to improve the elastic property of Portland cement paste (PC) and explain the interaction mechanism in the composites. A significantly improved elastic property that slightly affected the compressive strength could be accomplished. The formation and interaction mechanisms were well described using dielectric spectroscopy.

## 2. Experimental Details

### 2.1. Materials

Ordinary Portland cement ASTM type 1 was produced by Siam City Cement Public Company Limited (Saraburi, Thailand). High-ammonia natural rubber latex (NL) was produced by Thai Rubber Latex Group Public Company Limited (Samut Prakan, Thailand). The chemical analysis of the NL is shown in Table 1. The commercial product cetyl trimethyl ammonium bromide (CTAB) was produced by Solabio Life Science (Beijing, China) (CAS-57-09-0).

### 2.2. Mixture Design

In order to study the effect of NR latex and CTAB on the cement paste and cement mortar properties, four groups of cement mixtures were prepared. The mixing ratios of the cement pastes are shown in Table 2 and Table 3, respectively. They were prepared with water-to-cement (W/C) ratios of 0.45. Then, 0%, 5%, 10%, and 15% NL were mixed into the cement paste and cement mortar. The CTAB was varied at different CTAB/C ratios, 0.125%, 0.25%, 0.5%, 1%, and 2%, respectively. PC was the control paste.

### 2.3. Mixing Process

The specimens were prepared in a stainless steel bowl. The raw materials were mixed according to ASTM-C192 [42]. First, the NL, CTAB, and water were stirred for 1 min under a slow stirring rate to avoid the formation of air bubbles. Second, the cement powder was added and 30 s allowed for the adsorption of the water. Third, the mixture was stirred for 2 min with a stirring rate of 200 rpm and vibrated for 10 s. Then, the paste was poured into molds of 25 × 25 × 25 mm for the compressive test (ASTM C109) [43] and the cylindrical molds of 23.5 mm in diameter and 47 mm in height for the tensile test (ASTM C496) [44]. After that, they were left to set and wrapped with cling film. Finally, after 24 h, the specimens were de-molded and cured in a curing box at room temperature, 25 ± 3 °C, humidity 95%, for 7 d and 28 d.

### 2.4. Characterization and Test Methods

The characterizations and properties (compressive, tensile strengths, and dielectric properties) of the (CTAB + NL)/PC composites were performed after 28 d of curing under water. Before the samples were tested, the samples were soaked in acetone to prevent cement hydration. The physical, mechanical, and dielectric properties were investigated. The X-ray diffractometry (XRD, PANalytical, EMPYREAN) (Shanghai, China) technique was used to characterize the phase compositions of the cement pastes in the 2θ range of 10–80°. The morphologies and microstructure were revealed using scanning electron microscopy (SEM) and energy dispersive spectrometry (EDX) (SEC, SNE–4500 M and LEO, 1450 VP) (Suwon, Korea). The dielectric properties of the PC, CTAB/PC, and (CTAB + NL)/PC composites were measured using KEYSIGHT E4990A Impedance Analyze (Santa Rosa, CA, USA) in the frequency range of 40–10^7^ Hz at 25 °C using an oscillation voltage of 0.5 V. Silver paint was used as an electrode material. Before the dielectric measurements, the samples were heated in an oven at 100 °C for 24 h. The dried samples were coated with silver paint on both sides to fabricate a simple parallel plate capacitor. The capacitance (C_p_) and dissipation factor (*d*) or loss tangent (tanδ) were corrected. The dielectric constant (ε′) was calculated from the C_p_ value, following the equation, ε′ = [C_p_*d*/(ε_0_)*A*], where *d* and *A* are the sample thickness and electrode area, respectively. ε_0_ is the permittivity of the free space (8.854 × 10^−12^ F/m).

The compressive strength test was performed in accordance with ASTM C109 [43]. Cube specimens, which were aged for 28 d, were tested. A tensile strength test was performed for the cylinder specimen (28 d). The measurements were performed in accordance with ASTM C496 [44] following Equation (1),

(1) $$f=\frac{2F}{\pi dl}$$

where $f\left(N\cdot {\mathrm{mm}}^{2}\right)$ is the tensile strength, F(N) is the breaking load, d (mm) is the diameter of the cylinder specimen, and l (mm) is the length of the specimen.

## 3. Results and Discussion

The effects of the combination of the CTAB and NL content on the density, compressive strength, tensile strength, and dielectric properties of the (CTAB + NL)/PC composites were studied by varying the CTAB/cement and NRL/cement ratios. All mix proportions were prepared at a fixed water/cement (W/C) ratio of 0.45.

### 3.1. Density and Microstructure of NL/PC Composites

**Figure 1:**
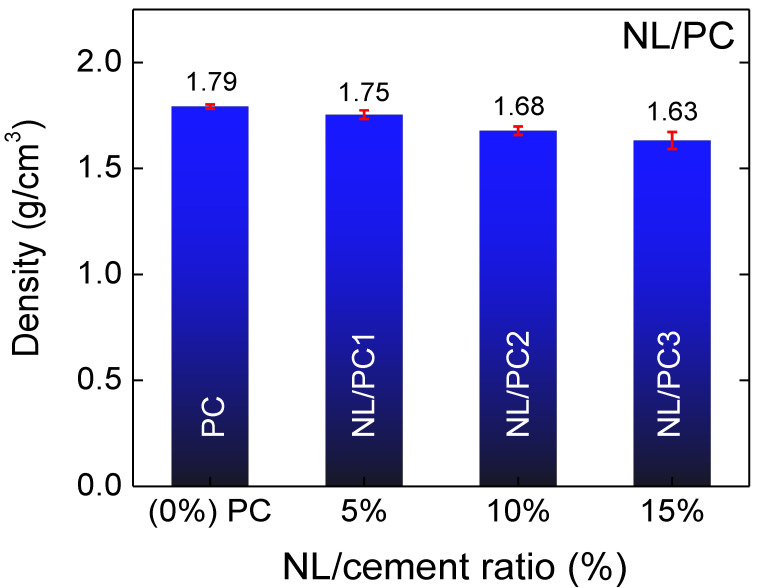
Bulk densities of the control PC and NL/PC1, NL/PC2, and NL/PC3 composites with NL/cement ratios of 5%, 10%, and 15%, respectively.

Figure 1 shows the bulk density dependence of the NL/C ratio for the NL/PC composites, which were cured for 28 d. The density of the PC was 1.79 g/cm^3^. The densities of the NL/PC composites with NL/cement ratios of 5% (NL/PC1), 10% (NL/PC2), and 15% (NL/PC3) were 1.75 g/cm^3^, 1.68 g/cm^3^, and 1.63 g/cm^3^, respectively. The density of the NL/PC composites slightly decreased with the increasing NL/C ratio. The densities were slightly reduced by 2.14%, 6.39%, and 8.93%, respectively, compared to that of the PC without the addition of NL. This observation was similar to that in the literature for the cement composites filled with NL [24].

**Figure 2:**
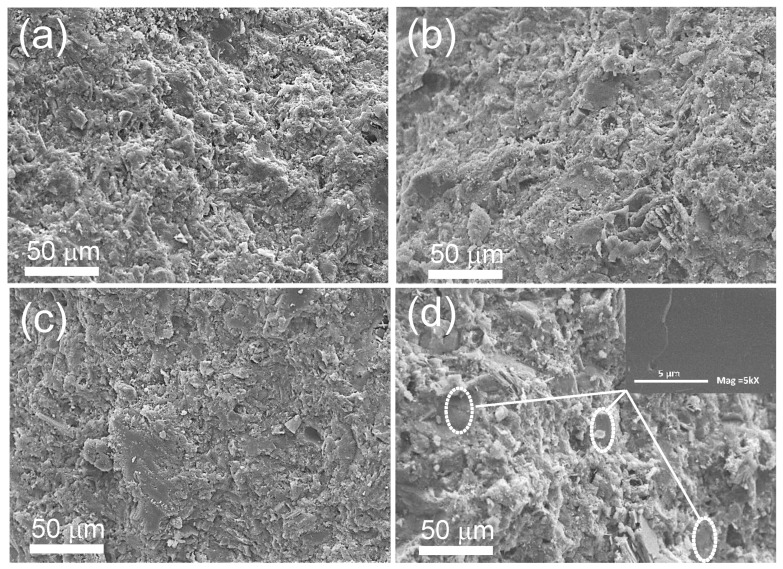
SEM images of the cross-section of the (**a**) control PC and (**b**) NL/PC1, (**c**) NL/PC2, and (**d**) NL/PC3 composites with NL/cement ratios of 5%, 10%, and 15%, respectively. The inset of (**d**) shows the flat, smooth surface of the NL.

Figure 2 shows the morphologies of the NL/PC composites with different NL/C ratios. No pores and a dense microstructure can be observed in all the composite samples. Thus, the reduced density of the NL/PC composites should not be associated with the pores. Instead, the decreased density was primarily attributed to the low specific gravity (0.946) of NL compared to that of PC (3.15). The rough surface of the significant component was observed for all the samples, indicating the PC phase. As shown in the inset of Figure 2d, a flat, smooth surface can be observed, indicating the separation of the NL phase.

### 3.2. Density and Microstructure of CTAB/PC and (CTAB + NL)/PC Composites

**Figure 3:**
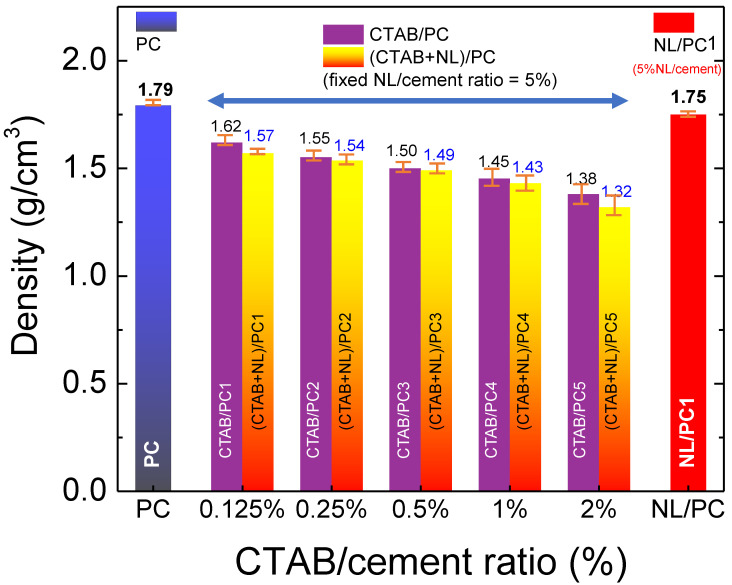
Bulk densities of CTAB/PC and (CTAB + NL)/PC (fixed NL/cement ratio = 5%) composites with different CTAB/cement ratios (0.125–2%) compared to those of the control PC and NL/PC1 composite.

The bulk density dependence of the CTAB/cement ratio for the CTAB/PC, which was cured for 28 d, is shown in Figure 3. The densities of the CTAB/PC with the CTAB/C ratios of 0.125% (CTAB/PC1), 0.25% (CTAB/PC2), 0.5% (CTAB/PC3), 1.0% (CTAB/PC4), and 2% (CTAB/PC5) were 1.62 g/cm^3^, 1.55 g/cm^3^, 1.50 g/cm^3^, 1.45 g/cm^3^, and 1.38 g/cm^3^, respectively. By comparing with the control PC sample, the densities of the CTAB/PC were reduced by 9.5–23.0%. Generally, the incorporation of CTAB can cause air bubbles, leading to a decrease in the densities of the CTAB/PC. Nevertheless, the densities of the CTAB/PC were still high, which were reduced by less than 14% compared to that of the control PC.

The objective of this study was to enhance the compatibility between the NL and PC using CTAB as a surfactant to improve the stability and workability of the PC. Thus, the (CTAB + NL)/PC composites were fabricated using different NL/cement and CTAB/cement ration, as summarized in Table 2 and Table 3. We found that the density of the (CTAB + NL)/PC composites was significantly decreased when the NL/cement ratio > 5.0%. Thus, the (CTAB + NL)/PC composites with an NL/cement ratio of 5.0% were further characterized by considering the variation in the CTAB/cement ratios. As shown in Figure 3, the densities of the (CTAB + NL)/PC1, (CTAB + NL)/PC2, (CTAB + NL)/PC3, (CTAB + NL)/PC4, and (CTAB + NL)/PC5 composites were 1.57 g/cm^3^, 1.54 g/cm^3^, 1.49 g/cm^3^, 1.43 g/cm^3^, and 1.32 g/cm^3^, respectively. Notably, the density of the CTAB/PC was slightly reduced by incorporating the NL. By comparing with the CTAB/PC, the density of the (CTAB + NL)/PC composites with the CTAB/C ratios < 0.1 was reduced by <3.1%.

**Figure 4:**
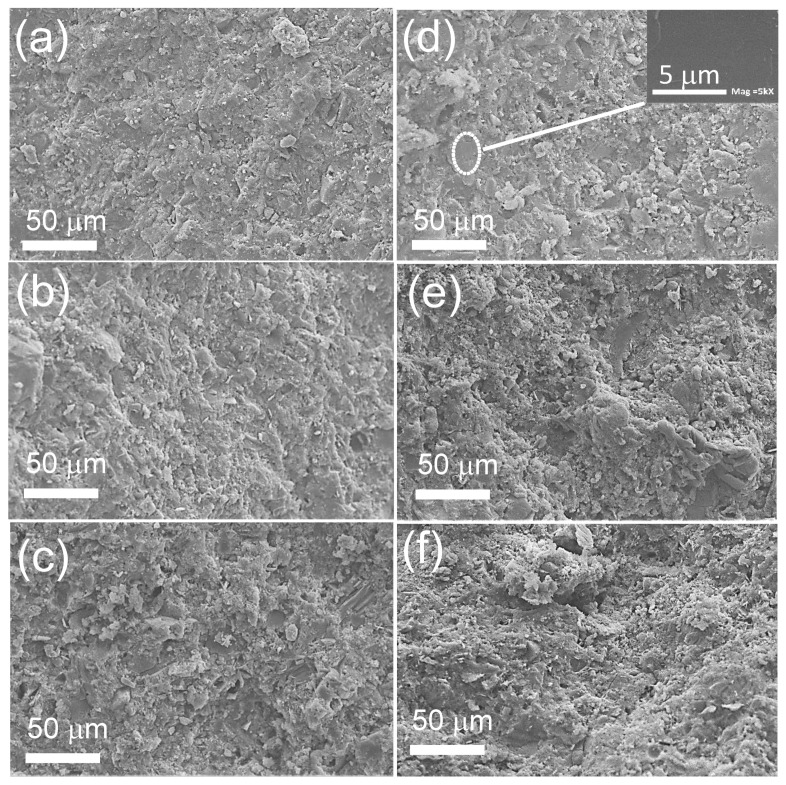
SEM images of the cross-sections of the (**a**) CTAB/PC1, (**b**) CTAB/PC2, (**c**) CTAB/PC3, (**d**) (CTAB + NL)/PC1, (**e**) (CTAB + NL)/PC2, and (**f**) (CTAB + NL)/PC3 composites. The inset of (**d**) shows the flat, smooth surface of the NL.

Figure 4 shows the morphologies of the CTAB/PC and (CTAB + NL)/PC (fixed NL/cement ratio = 5.0%) composites with various CTAB/cement ratios. As expected, pores cannot be observed obviously. The reduced densities of the CTAB/PC and (CTAB + NL)/PC composites were therefore attributed to the low specific gravities of the CTAB and NL. The rough surface of the major component was observed for all the samples, indicating the PC phase. As shown in the inset of Figure 4d, flat, smooth surfaces were observed, confirming the existence of the NL phase.

### 3.3. Phase Formation of PC and (CTAB + NL)/PC Composites

**Figure 5:**
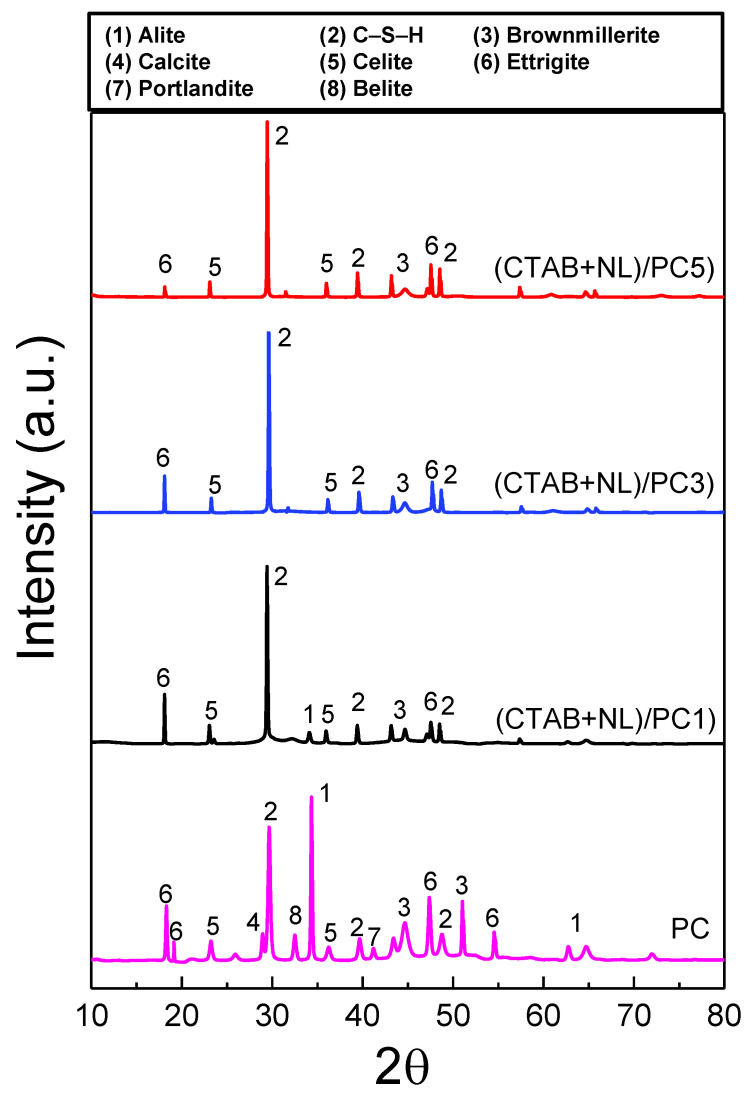
XRD patterns of the PC and (CTAB + NL)/PC1, (CTAB + NL)/PC3, and (CTAB + NL)/PC5 composites after 28 d of curing time.

Figure 5 represents the XRD patterns of the PC and the (CTAB + NL)/PC composites. The PC contained major crystalline phases such as alite (Peak No. 1), belite (Peak No. 8), brownmillerite (Peak No. 3), calcite (Peak No. 4), portlandite (Peak No. 6), ettringite (Peak No. 5), and calcium silicate hydrates (CSH (Peak No. 2)) [17]. Generally, the alite, belite, and brownmillerite phases are found in Portland cement [45]. After 28 d of hydration, the characteristic intensity of the belite and alite (around 33° and 34°) were observed in the PC, but they were not observed in the (CTAB + NL)/PC composites. The characteristic peaks of the portlandite, ettringite, and CSH were also observed in the PC and (CTAB + NL)/PC composites after 28 d of hydration. The explanation for this is that both alite and belite react with water, which produces CSH and portlandite, as shown in Equations (2) and (3) [46].

(2) $$\underset{\left(\mathrm{Alite}\right)}{2(3\mathrm{CaO}.{\mathrm{SiO}}_{2})}+\underset{\left(\mathrm{Water}\right)}{6H_{2}O}\to \underset{\left(C-S-H\right)}{3\mathrm{CaO}.2{\mathrm{SiO}}_{2}3H_{2}O}+\underset{\left(\mathrm{Portlandite}\right)}{3\mathrm{Ca}{\left(\mathrm{OH}\right)}_{2}}$$

(3) $$\underset{\left(\mathrm{Belite}\right)}{2(2\mathrm{CaO}.{\mathrm{SiO}}_{2})}+\underset{\left(\mathrm{Water}\right)}{4H_{2}O}\to \underset{\left(C-S-H\right)}{3\mathrm{CaO}.2{\mathrm{SiO}}_{2}}+\underset{\left(\mathrm{Portlandite}\right)}{\mathrm{Ca}(\mathrm{OH}{)}_{2}}$$

According to Equations (1) and (2), during hydration, the alite and belite phases of the cement paste convert to hydration products, including CSH and portlandite. The intensity of these peaks can be utilized to evaluate the effect of the CTAB/cement ratios on the cement hydration reactions. It is noteworthy that the belite, calcite, and portlandite peaks were not detected in the (CTAB + NL)/PC composites. There was a decrease in the alite peaks. When the CTAB/cement ratios increased, the alite peak was not observed ((CTAB + NL)/PC3 and (CTAB + NL)/PC5). On the other hand, the increased intensity of the CSH peak was observed. It is possible that the addition of CTAB resulted in a supported hydration process. The portlandite phase was not observed, which may be due to, but not prove, the reaction between CTAB and Ca(OH))_2_. The disappearance of portlandite corresponds to the disappearance of the calcite phase. The calcite peak was not observed in the (CTAB + NL)/PC composites. Generally, the calcite crystal can be formed by the reaction of airborne carbon dioxide and portlandite, which involves the diffusion of CO_2_ into cement specimens, as the following equation;

(4) $$\underset{\left(\mathrm{Portlandite}\right)}{{\mathrm{Ca}(\mathrm{OH})}_{2}}+{\mathrm{CO}}_{2}\to \underset{(\mathrm{Calcite})}{{\mathrm{CaCO}}_{3}}+H_{2}O$$

The continued carbonation of cement paste can be deteriorative and must be controlled. It is possible that the (CTAB + NL)/PC composites exhibited higher impermeability of CO_2_, which can reduce the availability of CO_2_ in the cement paste and thus improve the carbonation resistance of the hardened cement paste. Therefore, the portlandite content was reduced in the (CTAB + NL)/PC composites. The following mechanism was proposed to explain the observed changes of the portlandite peaks. At high pH values of fresh cement paste and NL, the carboxylic group of CTAB is ionized. Carboxylic ions then absorb divalent calcium ions from the cement hydrates and reduce the portlandite content of the hydrated cement paste [17]. Generally, the role of NL has a complicated effect on cement hydration, depending on the type of NL the, curing time, the curing conditions, and other factors. Hydration would slow down by forming latex films on the surface of cement particles, which reduces the contact area of the cement grains and water [47]. In this study, CTAB was used as a surfactant to improve the workability and solve the above problem. CTAB can exist on the surface of the NL particles, which may absorb water and also prevent the evaporation of water during the curing period. Moreover, the water absorption of CTAB leads to saving water inside the cement and supplying the required water for the hydration of cement over long time periods.

### 3.4. FTIR Spectroscopy

**Figure 6:**
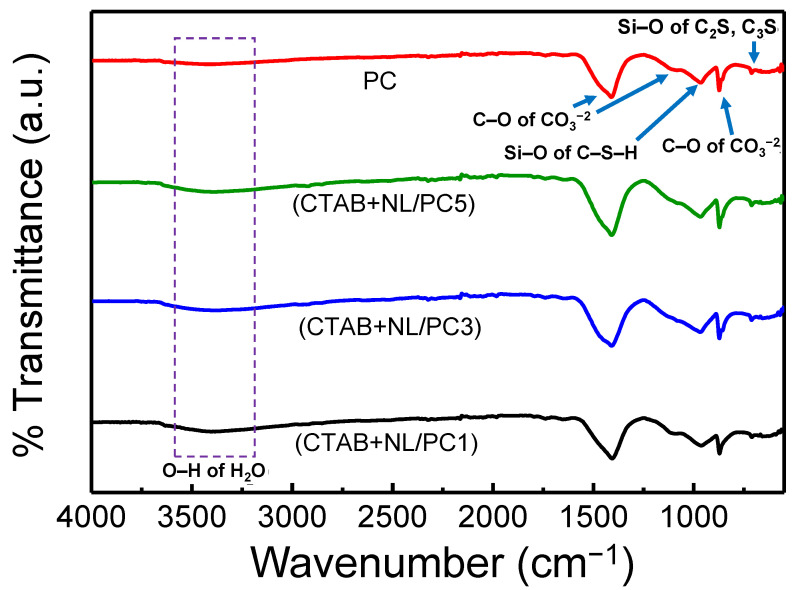
FTIR spectra of the control PC and (CTAB + NL)/PC1, (CTAB + NL)/PC3, and (CTAB + NL)/PC5 composites after 28 d of curing time.

Figure 6 displays the FTIR spectra of the PC and (CTAB + NL)/PC composites, which were cured at 28 d. The broad peaks in the range 3200–3600 cm^−1^ were associated with the O–H in the H_2_O of the hydrating products. Additionally, the peaks in the rage of 1408–1410 cm^−1^ and 873 cm^−1^ may be ascribed to the stretching of CO_3_^2−^ in the cement paste. These peaks in the (CTAB + NL)/PC composites at 1410 cm^−1^ slightly shifted towards higher wavenumbers when compared with the PC at 1408 cm^−1^. This may be due to the interaction among the cement, NL, and CTAB. A previous study reported that the IR peak of CTAB (1410–1416 cm^−1^) is the C–H stretching of the organic molecules of CTAB [1]. The band at around 965 cm^−1^ may be ascribed to a calcium silicate hydrate phase (C–S–H) [23,47], as seen in the PC, (CTAB + NL)/PC1, (CTAB + NL)/PC3, and (CTAB + NL)/PC5 composites. These findings supported the XRD results, indicating C–S–H, which was formed during the hydration of the cement according to Equation (3).

### 3.5. Compressive Strength, Tensile Strength, and Optimized Mechanical Performance

**Figure 7:**
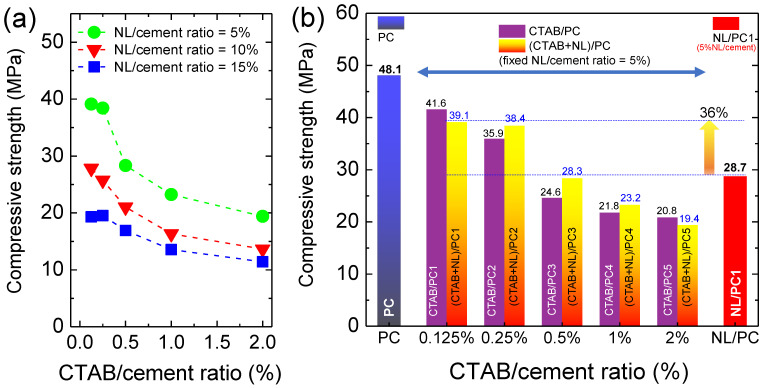
(**a**) CTAB/cement ratio dependence of the compressive strength for the (CTAB + NL)/PC composites with NL/cement ratio = 5%, 10%, and 15%. (**b**) Compressive strength of the CTAB/PC and (CTAB + NL)/PC (fixed NL/cement ratio = 5%) composites with different CTAB/cement ratios (0.125–2%) compared to those of the control PC and NL/PC1 composite.

Figure 7a shows the CTAB/cement ratio dependence of the compressive strength of the (CTAB + NL)/PC composites with different NL/cement ratios (i.e., 5%, 10%, and 15%). As can be clearly seen, the compressive strength slightly decreased when the CTAB/cement ratio was increased from 0.125% to 0.25%. After that, the compressive strength significantly decreased as the CTAB/cement ratio further increased. Considering any CTAB/cement ratio, the compressive strength of the (CTAB + NL)/PC composites decreased with the increasing NL/cement ratio from 5% to 15% even at a low CTAB/cement ratio (0.125–0.25%). This result is similar to those reported in the literature [24]. This is directly related to the significantly decreased density of the (CTAB + NL)/PC composites due to the presence of voids as the NL content increased. Furthermore, NL films can cause the retardation effect [12], leading to decreased compressive strength. Thus, we focused on the (CTAB + NL)/PC composites with the NL/cement ratio of 5%. Hopefully, the introduction of CTAB may enhance the mechanical properties of the NL/PC composites.

Figure 7b shows the compressive strength of the PC, NL/PC, and (CTAB + NL)/PC composites with different CTAB/cement ratios after a curing time of 28 d. First of all, it was found that the compressive strength of the NL/PC1 (28.7 MPa) was remarkably decreased by up to 40% compared to that of the control PC (48.1 MPa). This result may not be primarily associated with the density since the density of the NL/PC1 was slightly lower than that of the control PC (Figure 1). Instead, the primary effect was due to the retardation effect [12]. The compressive strength of the CTAB/PC decreased with increasing the CTAB/cement ratio from 0.125% to 2%. This result was directly correlated with the decrease in the decreased density due to air bubbles from the CTAB (Figure 3). Notably, the compressive strength of the CTAB/PC with CTAB/cement ratios ≤ 0.25 was reduced by less than ~25% only. Surprisingly, the compressive strength of the NL/PC1 (NL/cement ratio = 5%) could be significantly increased from 28.7 MPa to 39.1 MPa by ~36% after the addition of the 0.125% CTAB/cement ratio, i.e., CTAB + NL/PC1. After that, the compressive strength of the (CTAB + NL/PC) composites slightly decreased with increasing the CTAB/cement ratio from 0.125% to 0.025%. This finding indicated that the compressive strength of the NL/PC composites can be effectively improved by adding a small amount of CTAB (≤0.25%).

**Figure 8:**
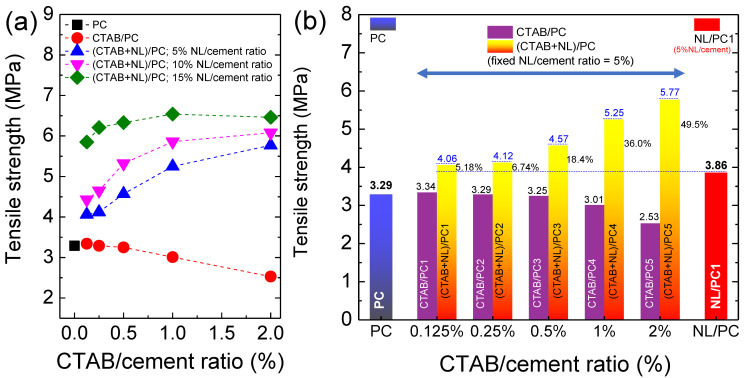
(**a**) CTAB/cement ratio dependence of the tensile strength for the CTAB/PC and (CTAB + NL)/PC composites with NL/cement ratio = 5%, 10%, and 15% compared to that of the control PC. (**b**) Tensile strength of the CTAB/PC and (CTAB + NL)/PC (fixed NL/cement ratio = 5%) composites with different CTAB/cement ratios (0.125–2%) compared to those of the control PC and NL/PC1 composite.

The effect of the CTAB and NL on the tensile strength of the PC is shown in Figure 8a. The tensile strength of the PC slightly changed when incorporating CTAB with the CTAB/cement ratio ≤ 0.5%, while their compressive strength decreased (Figure 7). Although the tensile strength of the NL/PC1, NL/PC2, and NL/PC3 composites could, respectively, be significantly improved to 3.86 MPa, 4.73 MPa, and 6.21 MPa, their compressive strength values were significantly reduced. Similarly, the tensile strength of the (CTAB/NL)/PC composites increased with increasing the NL/cement ratio. As demonstrated in Figure 8b, the tensile strength values of all the (CTAB + NL)/PC composites were higher than those of the control PC, CTAB/PC, and the NL/PC1 composite. Notably, the tensile strength of the NL/PC composites could be significantly increased by incorporating CTAB. When the content of CTAB ≤ 0.5%, the CTAB not only had no effect on the degradation of the tensile strength of the PC, but also enhanced the tensile strength of the NL/PC composites. This result indicated that the CTAB plays some role in interacting with the NL and PC matrix.

Although the tensile strength of the NL/PC composites could be significantly increased, the compressive strength was greatly reduced. While the compressive strength of the CTAB/PC was not largely decreased, the tensile strength was not significantly improved. On the other hand, although the compressive strength of the (CTAB + NL)/PC composites decreased, the tensile strength significantly increased compared to that of the PC. Due to both the tensile and compressive strengths, which generally tend to change in the opposite way, the optimization of these two parameters should be studied. The optimized compressive–tensile performance (OCTP) is proposed to show an overall view, as expressed by Equation (5),

OCTP = [Tensile strength × Compressive strength](5)

**Figure 9:**
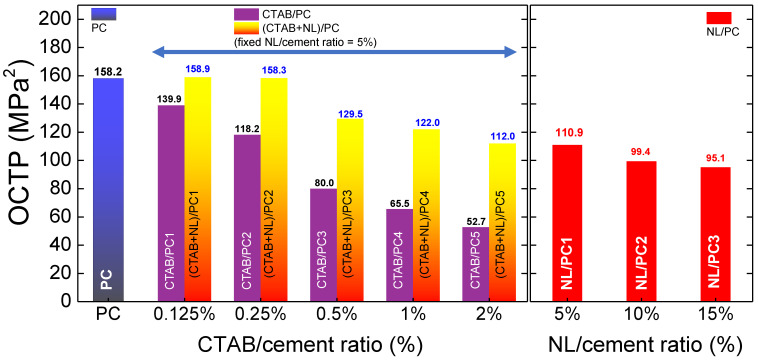
Optimized mechanical performance of the PC, CTAB/PC, NL/PC, and (CTAB + NL)/PC composites.

The calculated results are demonstrated in Figure 9. Obviously, the OCTPs of the CTAB/PC and NL/PC composites were significantly lower than that of the PC. Interestingly, the *OCTPs* of the (CTAB + NL)/PC composites with the NL/cement ratio of 5% and CTAB/cement ratios of 0.125% and 0.25% were nearly equal to that of the PC. As shown in the SEM images, some large clusters of CTAB + NL on the PC were observed. Usually, the dispersion degree of additives plays an important role in influencing the mechanical performance of PC [48]. Thus, this observation may be also an important cause of the observed decrease in the mechanical performance of the PC.

**Figure 10:**
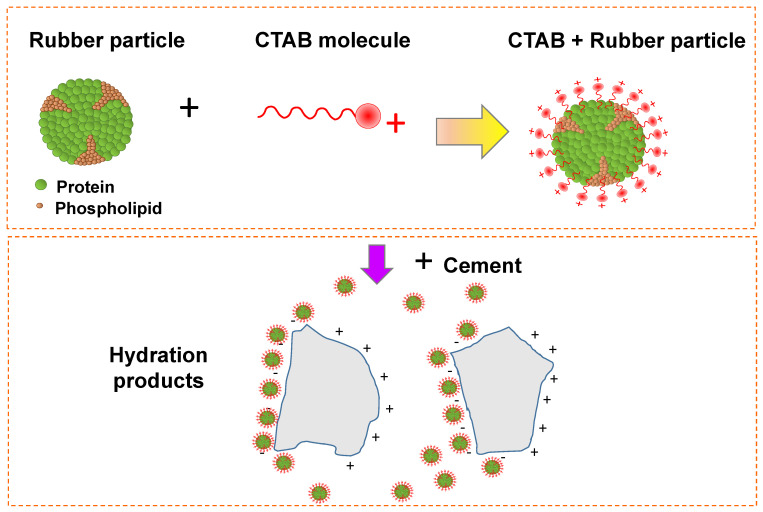
Schematic of the possible mechanism of the interaction between the NL–CTAB–PC matrix.

We now turn to explain the possible mechanism of the interaction between NL–CTAB–PC matrix. Generally, the important role of CTAB in the preparation of nanoparticles is to adsorb onto the surface of the nanoparticles, giving a decreased surface energy [49]. Thus, CTAB can prevent the aggregation or agglomeration of nanoparticles. As shown in Figure 10, the structure of the NL particle consists of a phospholipid–protein shell and a polyisoprene core [17,50]. The hydrophobic surface of NL particles adsorbed the tail of the CTAB by hydrophobic interaction [51]. The CTAB molecules enclosed the NL particles, giving rise to the positively surface-changed particles. In this case, the NL particles were stabilized by the cationic part of the CTAB. Thus, the agglomeration of the NL particles was inhibited by the repulsive forces between the positively surface-charged particles. As a result, the homogeneous dispersion of the NL particles in the PC matrix was expected. The aluminate phases such as ettringite, C_4_AF, and C_3_A are classified as the positively charged phase, while the negatively charged phases, i.e., silicate phases, consist of CeSeH, C_2_S, and C_3_S [47,52]. Thus, the positively charged particles of the NL can interact with the negatively charged phases in the PC matrix by the Coulomb force. The enhanced mechanical properties were obtained by this mechanism. Besides the PC matrix composites, CTAB is generally applied as a common surfactant to reinforce the incompatibility of two or more components. Chhetri et al. [53] reported the improvement of the mechanical properties of MoS_2_/epoxy composites using CTAB as a surfactant. At 0.2 wt% CTAB-MoS_2_ loading, the tensile strength could be enhanced by ~23%. Furthermore, the tensile strength of the polyphenylene sulfide could be increased by ~45.5% after incorporating CTAB-graphene oxide [54].

### 3.6. Dielectric Properties

**Figure 11:**
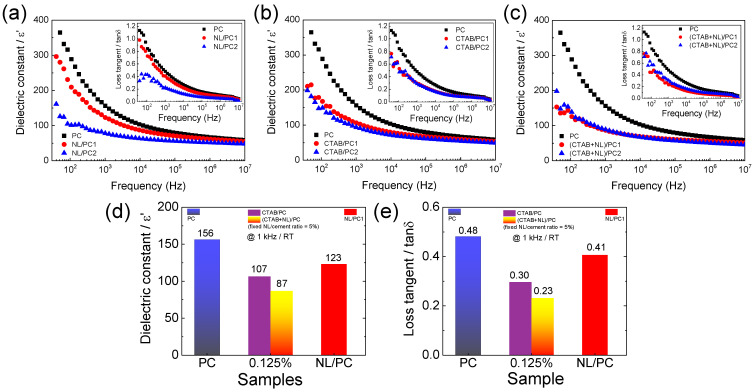
Frequency dependence of the dielectric constant (ε′) at 25 °C for the (**a**) NL/PC, (**b**) CTAB/PC, and (**c**) (CTAB + NL)/PC composites compared to that of the PC; the insets show the dielectric loss tangent (δ) at 25 °C. (**d**,**e**) Comparison of ε′ and tanδ for the PC, CTAB/PC1, NL/PC1, and (CTAB + NL)/PC1 composites.

Figure 11 shows the dielectric properties at 25 °C for the PC, CTAB/PC, NL/PC, and (CTAB + NL)/PC composites in the temperature range of 40–10^7^ Hz. As shown in Figure 11a–c and the insets, the ε′ and tanδ values of the PC decreased by incorporating NL, CTAB, and (CTAB + NL) over the measured frequency range. The large decreases in the ε′ and tanδ values were obviously observed in a low-frequency range (<10^4^ Hz). Furthermore, the frequency-dependent behaviors of the ε′ and tanδ values for all the samples were similar. The ε′ and tanδ values decreased with increasing frequency. When the frequency increased, the dipole moments in the samples had difficulty rotating following the direction of an applied AC field, giving rise to the decreased polarization in the samples and, hence, the ε′ value [40,55,56,57]. Generally, energy loss in an insulator is caused by the dielectric relaxation process in a high-frequency range and the long-range motion of free charge carriers (i.e., DC conduction) [39,40]. The total loss tangent in a low-frequency range was primarily attributed to the DC conduction, which can be estimated as the following relationship [40,41],

(6) $$\tan\delta \approx \frac{\sigma_{dc}}{2\pi f\varepsilon_{0}\varepsilon_{s}^{\prime }}\approx {\left(2\pi f\varepsilon_{0}\varepsilon_{s}^{\prime }\rho_{dc}\right)}^{-1},$$

where $\varepsilon_{s}^{\prime }$ is the static dielectric constant, *f* is the frequency of an applied AC field, and *σ_dc_* and *ρ_dc_* are the DC conductivity and resistivity, respectively. Thus, the tanδ in a low-frequency range would be decreased with increasing frequency. Besides the conductivity, other possible contributions to the tanδ value such as the electrode polarization, impurities on the surface, and various defects on the surface sample may have effects on the low-frequency tanδ value.

Figure 11d,e shows the comparisons of the ε′ and tanδ values at 1 kHz for the PC, CTAB/PC-1, NL/PC1, and (CTAB + NL)/PC1 composites. The ε′ and tanδ values of the NL/PC1 (ε′ = 123, tanδ = 0.41) were lower than those of the PC (ε′ = 156, tanδ = 0.48). According to the raw mixing of the dielectric composite [37,38], this observation was caused by a low ε′ value (~2–3) of the NL filler [58,59,60], which was incorporated in the PC matrix. The reduced tanδ value was also due to the low tanδ of the NL (<0.01), which was due to the large resistivity [59,61,62].

Furthermore, the ε′ and tanδ values of the CTAB/PC1 were also lower than those of the PC. In this case, the decreased ε′ value cannot be explained using the mixing law of the composite because CTAB is the molecule. As demonstrated in Figure 10, the positive charge of CTAB interacted with the negatively charged phases in the PC matrix. Thus, the free charges in the PC were reduced. Generally, a low-frequency dielectric response in heterogeneous materials is primarily originated from the interfacial polarization [36,40]. In this case, negative and positive free charges were separated and accumulated at the opposite side of the discontinuous phases, giving rise to the interfacial polarization. Therefore, the interfacial polarization would be decreased by decreasing the free charge carriers [39]. This was the primary cause of the observed decrease in the ε′ value of the CTAB/PC. According to Equation (6), the tanδ value is dependent on σ_dc_, which is inversely proportional to ρ_dc_. Thus, the decrease in the free charges in the CTAB/PC1 not only resulted in a decreased ε′ value, but also a decrease in tanδ. It is worth noting that the decreased ε′ values of the NL/PC and CTAB/PC were also caused by the decrease in their densities. Interestingly, the ε′ and tanδ values of the (CTAB + NL)/PC1 were the lowest among all the samples. This result was due to the combination effects in the NL/PC and CTAB/PC. The decreased ε′ value was due to the low ε′ of the NL and the reduced free charges due to the Coulomb interaction between NL–CTAB–PC, while the decrease in the tanδ value was associated with the excellent insulator of the NL and the reduced free charges. The dielectric results supported the hypothesis that there was strong interaction between the NL–CTAB–PC, leading to the enhanced mechanical properties of the (CTAB + NL)/PC composites.

## 4. Conclusions

In conclusion, we successfully fabricated natural rubber latex/cement paste composites with improved elastic properties using CTAB as a surfactant to enhance the stability and workability of the NL particles and the PC matrix without anti-foaming admixture. The existence of the NL in the PC matrix was revealed using the SEM technique. The study of the phase formations showed that the portlandite phase in the (CTAB + NL)/PC composites disappeared, giving rise to a decrease in the degradation process. The tensile strength of the (CTAB + NL)/PC was significantly increased from 3.29 MPa (PC) to 4.12 MPa, while the compressive strength was slightly decreased from 48.08 MPa to 38.42 MPa for the (CTAB + NL)/PC composite using the NRL/C and CTAB/C ratios of 5% and 0.25%, respectively. The ε′ and tanδ values of the NL/PC, CTAB/PC, and (CTAB + NL)/PC were reduced compared to that of the PC, which was caused by the low ε′ value of the NL filler and the ability of the CTAB to trap negative changes in the PC, leading to the decreased interfacial polarization and, hence, ε′. This work provides a comprehensive guideline for significantly improving the elastic property of PC while retaining a high compressive strength of PC.

## Figures and Tables

**Table 1:** Chemical analysis of the NL.

Property	Test Results
Total solid content	61.74%
Dry rubber content	60.12%
Non-rubber content	1.62%
Alkalinity (as ammonia by total weight)	0.69%
Volatile fatty acids	0.0282%
pH value at 25.6	10.49
Mechanical stability time	800 s
Specific gravity at 25 °C	0.9456

**Table 2:** Mixing ratios for cement pastes with a W/C ratio of 0.45.

Samples	NL/Cement Ratio (%)	CTAB/Cement Ratio (%)
PC	-	-
NL/PC1	5	-
NL/PC2	10	-
NL/PC3	15	-
CTAB/PC1	-	0.125
CTAB/PC2	-	0.25
CTAB/PC3	-	0.50
CTAB/PC4	-	1.00
CTAB/PC5	-	2.00
(CTAB + NL)/PC1	5	0.125
(CTAB + NL)/PC2	5	0.25
(CTAB + NL)/PC3	5	0.50
(CTAB + NL)/PC4	5	1.00
(CTAB + NL)/PC5	5	2.00
(CTAB + NL)/PC6	10	0.125
(CTAB + NL)/PC7	10	0.25
(CTAB + NL)/PC8	10	0.50
(CTAB + NL)/PC9	10	1.00
(CTAB + NL)/PC10	10	2.00
(CTAB + NL)/PC11	15	0.125
(CTAB + NL)/PC12	15	0.25
(CTAB + NL)/PC13	15	0.50
(CTAB + NL)/PC14	15	1.00
(CTAB + NL)/PC15	15	2.00

**Table 3:** Mixing proportions of cement pastes with 1000 g of Portland cement.

Samples	CTAB Powder (g)	Natural Rubber Latex			Addition Water II(g)
		NL Content (g)	Water in NL (g)	Solid in NL (g)	
PC	-	-	-	-	450
NL/PC1	-	50	19	31	431
NL/PC2	-	100	37	62	412
NL/PC3	-	150	58	93	393
CTAB/PC1	1.25	-	-	-	450
CTAB/PC2	2.50	-	-	-	450
CTAB/PC3	5.0	-	-	-	450
CTAB/PC4	10.0	-	-	-	450
CTAB/PC5	20.0	-	-	-	450
(CTAB + NL)/PC1	1.25	50	19	31	431
(CTAB + NL)/PC2	2.50	50	19	31	431
(CTAB + NL)/PC3	5.0	50	19	31	431
(CTAB + NL)/PC4	10.0	50	19	31	431
(CTAB + NL)/PC5	20.0	50	19	31	431
(CTAB + NL)/PC6	1.25	100	38	62	412
(CTAB + NL)/PC7	2.50	100	38	62	412
(CTAB + NL)/PC8	5.0	100	38	62	412
(CTAB + NL)/PC9	10.0	100	38	62	412
(CTAB + NL)/PC10	20.0	100	38	62	412
(CTAB + NL)/PC11	1.25	150	57	93	393
(CTAB + NL)/PC12	2.50	150	57	93	393
(CTAB + NL)/PC13	5.0	150	57	93	393
(CTAB + NL)/PC14	10.0	150	57	93	393
(CTAB + NL)/PC15	20.0	150	57	93	393

## Data Availability

The data presented in this study are available on request from the corresponding author.
